# A guidebook describing the contribution Family Medicine can make in improving health systems

**DOI:** 10.4102/phcfm.v6i1.638

**Published:** 2014-07-24

**Authors:** Colette Gunst

**Affiliations:** 1 Division of Family Medicine and Primary Care, Stellenbosch University and Cape Winelands District, Department of Health, Western Cape Government, South Africa

It was with interest that I looked at this 280 page book written on a topic that is challenging to me as a practising Family Physician in South Africa, where Family Medicine has only recently been afforded the status of a specialist discipline. In my context the contribution of family medicine to management and improving health systems is not always seen as the main area of influence for a clinician. A quick flick through the pages of this softcover book, easy to hold (in the era of e-books some of us still love the feel of paper pages), showed that there were scenarios and examples from various countries interspersed amongst the rich text.

In the foreword by Dr Margaret Chan, Director-General of the World Health Organization (WHO), she gives her opinion that ‘this guidebook can help countries throughout the world maintain and improve health and well-being by developing a more productive, coordinated and cost-effective approach to health care’.

Chapter 1 starts with a summary of health care in the world. Family medicine serves to ‘link those concerned with population health and those who are at the forefront of delivering health care to individuals’. Family medicine is also clearly defined from the outset of the book and detail is provided of the range of contexts in which family doctors work, as well as their scope of practice, and details various scenarios to describe the spectrum of developmental stages at which countries are in implementing family medicine.

Chapter 2 provides the context for the rest of the book, by reviewing the values, goals and functions of health systems. Discussion in this chapter is very broad and quite idealistic as values and goals should be – how to achieve them in sub-Saharan Africa (SSA) with its variable social determinants of health, will be the challenge. Some ideas to address the limited human and physical resources in low-income countries and rural areas are provided. The pivotal role of Primary Health Care (PHC) is presented in detail, including the need for a focus on community oriented primary health through integrating clinical medicine with public health at community level.

Family doctors are seen as a critical link within the health system, and Chapter 3 details the nature of family medicine based on the World Organization of National Colleges, Academies and Academic Associations of General Practitioners and/or Family Physicians (WONCA) definition and what contribution family doctors can make to health care. A well-written and reflective account is given of the family doctor as an excellent clinician, working within a specific community, and addressing the patient’s needs within that community, with examples from around the world.

In SSA, where the formalisation of family medicine is still being achieved, the chapter on education and professional development in family medicine is good to read and consider how it can be adapted in our particular setting, to ensure that there is a primary health care focus in both under- and post-graduate training. Partnerships with medical schools and family medicine organisations, as well as learning lessons from other countries, becomes critical to ensure that a strong system of education and continuous professional development is implemented.

Necessary education and training prepares family doctors, whilst effective health systems and collaborative teams provide the organisational structure necessary for the delivery of efficient health services. Chapter 5 explores how to construct this supportive environment. After each key aspect is discussed, there is an ‘information box’ of practical strategies which the family doctor and manager can employ.

The final two chapters address issues particularly relevant to SSA. In the discussion on family medicine in lower-and upper-middle income countries, written by the WHO, case studies from Brazil, China, code with your smart phone or mobile device to read online. the Eastern Mediterranean region, and Thailand are provided. Although no African country is included in the case studies, the pivotal role of family medicine is reviewed. The challenge will be how we use the lessons provided to strengthen and ‘entrench’ the place of family medicine in our continent.

Finally, the development of family medicine in Africa in the twenty-first century is discussed – ‘the African family physician’. An interesting and detailed account of how the development of family medicine has been supported by WONCA and Flemish universities is provided; including the origin and purpose of the Primary care and/or family medicine education (Primafamed) network. One of the major achievements of the first Primafamed Conference in Kampala in 2008, was the launch of the open-access, online journal – the African Journal of Primary Health Care and Family Medicine.

The challenge of poverty, the ‘brain drain’, hospital versus community-focused care, the tension between horizontal and vertical care, are not new to those of us working in Africa; what is needed is a systematic approach to dealing with the challenges, so that health care to all (even those in the remotest remote areas) can become a reality. One strategy is to reach a ‘critical mass of well-trained family physicians… to demonstrate the contribution or family medicine to effective primary health care’. This book helps to guide managers, policy-makers, family physicians, educators, and ‘believers in the effectiveness of PHC’, on how to achieve this critical mass. It is a useful guidebook, with detailed reference lists, which I will keep on my desk to refer to as we plan to improve health systems in my own district in the Cape Winelands of the Western Cape.

